# The FareAssieme Model of Trento: Empowering Community Psychiatry through Peer Support Experts

**DOI:** 10.1192/j.eurpsy.2025.322

**Published:** 2025-08-26

**Authors:** W. A. R. Di Napoli, D. Scordato, O. Andreatta, S. Biasi, R. Pederzolli, C. Agostini

**Affiliations:** 1 Trento Mental Health Center, Azienda Provinciale per i Servizi Sanitari - Trento; 2 Il Cerchio Fareassieme, Trento, Italy

## Abstract

**Introduction:**

“FareAssieme” (“Doing Together”) is a community psychiatry approach adopted by the Mental Health Service of Trento (SSM) since 1999. It is based on the active involvement of users and families, valuing “experiential knowledge”—the insights gained from their experiences with illness and recovery—to enhance mental health care and rehabilitation pathways.

**Objectives:**

To describe the “FareAssieme” model and analyze the effectiveness of Peer Support Experts (ESPs) in recovery processes, stigma reduction, and improving patient-perceived quality. ESPs are users or family members who have achieved life balance in their recovery journey and developed coping skills, sharing their stories to support others within the SSM of Trento.

**Methods:**

The approach was implemented by systematically including ESPs in all SSM areas, such as front-office services, community mental health teams, territorial and hospital crisis areas, and residential facilities. Evaluation methods included satisfaction questionnaires, systematic PREMs and PROMs (Patient-Reported Experience and Outcome Measures), focus groups, and direct field observations, with particular attention to integrating ESPs into professional teams.

**Results:**

Introducing ESPs led to significant service quality improvements, increased active user involvement, and a better working climate. Patients reported greater trust in services and enhanced recovery experiences. ESPs helped overcome users’ initial resistance, especially among those less willing to trust health services, fostering stronger therapeutic relationships. Professional resistance diminished as the added value of ESPs became evident. The “FareAssieme” model also fostered the establishment of a Participatory Planning Group (GPP), a diverse team including ESPs and professionals. The GPP discusses relevant topics for the SSM’s operations and has produced several “Operational Guidelines”—recommendations compiled into booklets serving as operational instructions for all users, families, and service operators.

**Conclusions:**

The “FareAssieme” model in Trento represents an effective example of recovery-oriented community psychiatry. ESPs have uniquely contributed to improving perceived service quality and promoting a culture of respect and co-participation, reducing the stigma associated with mental illness. The GPP has also served as a representative for mental health issues, voicing the SSM’s shared views through mass media to disseminate anti-stigma messages.

**Disclosure of Interest:**

None Declared

